# Early Attachment and the Development of Social Communication: A Neuropsychological Approach

**DOI:** 10.3389/fpsyt.2022.838950

**Published:** 2022-04-08

**Authors:** Vibhuti Jethava, Jocelyn Kadish, Lisa Kakonge, Catherine Wiseman-Hakes

**Affiliations:** ^1^York Hills Centre for Children, Youth and Families, Richmond Hill, ON, Canada; ^2^Department of Speech-Language Pathology, University of Toronto, Toronto, ON, Canada; ^3^School of Rehabilitation Science, Speech Language Pathology Program, McMaster University, Hamilton, ON, Canada; ^4^KITE Research Institute, Toronto Rehab-University Health Network, Toronto, ON, Canada; ^5^Holland Bloorview Kids Rehabilitation Hospital, Toronto, ON, Canada

**Keywords:** social communication, social cognition, mental health early attachment and relationships, intervention, assessment neuropsychological approach, infant development

## Abstract

Social communication forms the foundation of human relationships. Social communication, i.e., the appropriate understanding and use of verbal and non-verbal communication within a social context, profoundly impacts mental health across the lifespan and is also highly vulnerable to neurodevelopmental threats and social adversities. There exists a strong interconnection between the development of language and other higher cognitive skills, mediated, in part, through the early attachment relationship. Consideration of how attachment links to brain development can help us understand individuals with social communication difficulties across the lifespan. The early attachment relationship supports the development of the foundational constructs of social communication. In this paper, a neuropsychological perspective was applied to social communication, which integrated evidence from early attachment theory, examining the underpinnings of social communication components identified by the SoCom model, namely socio-cognitive, socio-emotional, and socio-linguistic constructs. A neuropsychological perspective underscores the importance of interdisciplinary collaboration. This should also inform approaches to prevention, policy, intervention, and advocacy for individuals with or at risk for social communication impairments, as well as their families.

## Introduction

Social communication is vital for human relationships and mental health across the lifespan. Social communication refers to the appropriate understanding and use of verbal and non-verbal communication within a social context (1). It includes much more than knowledge of structural aspects of language (e.g., vocabulary and grammatical rules), but also the use of language as a way to engage socially with others. Disorders of Social Communication are associated with etiologies such as Autism Spectrum Disorder and Acquired Brain Injury, whereas developmental language disorders (DLD) refer to language impairments not associated with any known causal condition (2). There exists a strong interconnection between the development of language and other cognitive skills, such as attention, information processing and cognitive flexibility (3), which is mediated, in part, through the early attachment relationship. A consideration of how attachment influences brain development (4) can help us understand the foundational constructs of social communication. Moreover, the early attachment process exemplifies the dynamic interplay of neurobiological, social and cognitive factors in the development of social communication. A neuropsychological perspective, as originally proposed by Vygotsky (5) and Luria (6), and more recently by Tomas and Vissers (7), provides a valuable framework to understand the role of attachment in supporting the development of language, cognitive and emotional skills essential for social communication.

Converging lines of evidence from neuroscience and the social and cognitive sciences suggest that early relationships and experiences have the capacity to impact the neurodevelopment of social and cognitive skills to the extent they can affect life trajectories. The early conceptualization of this idea began “in the 1940’s” when Hebb first proposed a theory that early environmental enrichment could promote structural changes in the brain and thus enhance brain development (8). Recent research has extended these findings by investigating the biological mechanisms by which early psychosocial factors influence neuronal development (9–12). This evidence supports a neuropsychological perspective, whereby human development is shaped by the interaction of biology and social experience, rather than simply “unfolding” as a predetermined sequence of developmental stages (13).

Attachment theory, first espoused by John Bowlby, defined attachment as a neurobiological system that is the result of “serve-and-return” interactions between the infant and caregiver (4). Within the context of this paper, we refer to the primary caregiver as any familiar and consistently available adult (e.g., mother, father), who is primarily responsible for the infant’s need and care (13). Attachment theory considers the “serve and return” interactions between infant and caregiver to be crucial in shaping cognitive, social and emotional development. More specifically, the attachment relationship facilitates the ability to understand interpersonal behavior (14, 15), an understanding which emerges in infancy, beginning with shared joint attention, the early development of self-regulation, and the inception of the capacity for empathy, all of which are fundamental to social communication (3).

From birth, infants are neurologically primed for social communication and interaction with their primary caregivers (16, 17). A central tenet of attachment theory is that infants develop an internal working model (IWM), a mental representation of self and others, and this IWM governs significant social relationships across the lifespan (13, 18–20). While the IWM concept has received criticism for the breadth of the construct, as a “catch all” for linking research findings, it continues to be used as a metaphor for understanding the impact of attachment relationships, and is the subject of ongoing research (21). Infants who repeatedly experience responsive, reparative, consistent, warm and sensitive responses from their caregiver display a secure attachment style (22). Securely attached infants have an IWM developed through repeated positive interactions, which communicates to them that adults are generally a trustworthy and reliable source of comfort (22). Conversely, an insecure attachment style will develop when the attachment system does not function to regulate the infant’s emotional states (i.e., if the caregiver repeatedly misses or misinterprets the infant’s cues or responds inappropriately) (23). These mis-attuned interactions alter the genetic expression and neurogenesis of neural networks involved in self-regulation (17). Insecurely attached infants thus present with a heightened stress response and maladaptive emotions, which may lead to poor socio-emotional coping (24). It is the quality of emotion regulation provided by the caregiver that constitutes the essential difference between a secure versus insecure attachment (25).

**FIGURE 1:**
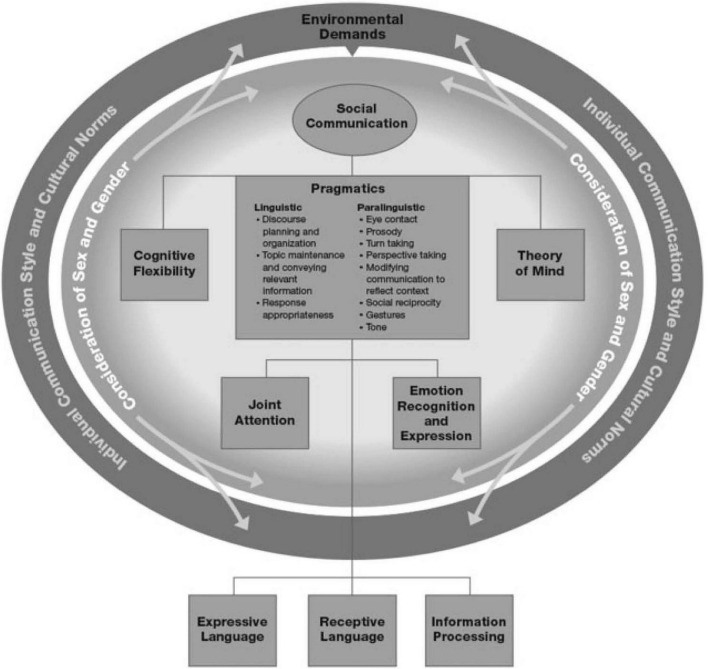
Social communication model (SoCom) (used with permission from the publisher, Georg Thieme Verlag KG).

Currently, there are a number of theoretical models of social communication, however, they vary in regard to the classification of components, inter-relationships and underlying processes (26). A recent model, the SoCom, developed by members of our group, provides a framework that identifies the cognitive, emotional and language based “building blocks” required for effective social communication (3) (see Figure 1). This paper will examine these component constructs through the lens of early attachment in the development of social communication.

Objectives: This paper aims to apply a neuropsychological perspective to social communication, examining the influence of the early attachment process on the development of foundational components of social communication. Consistent with the SoCom (3), these components include cognitive, emotional and linguistic. Given that these constructs are examined as underpinnings for social communication, we refer to them as “socio-cognitive,” “socio-emotional,” and “socio-linguistic” constructs.

### Developmental Neuropsychological Perspective

Human development is a dynamic product of the neurobiological system within the child interacting with systems of social contexts. The early attachment relationship and contexts are powerful drivers of human neuropsychological development and can be enriching and supportive or, conversely, a source of stress and risk (27). The attachment relationship serves as a template for the child’s subsequent relationships and influences the development of the following fundamental constructs underlying social communication: socio-cognitive, socio-emotional and socio-linguistic skills.

### Socio-Cognitive Development

Early caregiving has been identified as an important determinant in the development of self-regulation, a key component of executive functioning (EFs) (28, 29). EFs are a set of higher-order skills which support verbal and non-verbal thinking and behavior, in a goal directed, controlled and flexible manner (30). Working memory, inhibition and cognitive flexibility are the functions most reported as associated with social communication (28, 31), while the functions of organization, planning, self-control and generativity are also considered important for social communication (31). The development of EFs are highly influenced by linguistic and emotional scaffolding within the attachment relationship (32). When the infant experiences distress, the attuned caregiver consistently helps the infant to re-establish equilibrium by moving from a state of distress to a state of calm, thus supporting the beginning of self-regulation. These experiences of attunement are critical to synaptic formation in the prefrontal cortex, and attuned caregiver-infant interactions have been shown to increase frontal brain activity (8). Interactions characterized by sensitivity, parent talk about mental states, and support for autonomy are associated with the development of children’s inhibition, working memory and executive functions (8). Secure attachment thus promotes the development of self-regulatory executive skills supporting goal-directed behaviour, self-monitoring and impulse control (33).

Self-regulation and language serve as important tools in executive control and are interdependent; self-regulation ability has been shown to drive language development, and vice versa (34). Language facilitates self-regulation by serving as a cognitive tool for mental organization, mental representation, and behavioral planning (5, 34). Inner speech (IS), the process by which children gradually internalize overt speech to accompany their activity in a variety of cognitive tasks (e.g., “I need to wait my turn”), has been shown to play a key role in executive functioning (35). Thus, strengths in language are associated with delayed gratification in children (36). At the same time, self-regulation enables children to maximize language-learning opportunities. Increased inhibitory control supports attention and engagement, enabling children to absorb and learn from conversational interactions and form relationships. Further, cognitive flexibility allows for application of variable rules of language, such as multiple meaning words, and pragmatic rules associated with different contexts (34). There is thus an intimate interplay between attachment, language and EFs. The internalized social dialog, i.e., the dialog which reciprocally exists between the child and caregiver in a secure attachment, is theorized to play a key mediating role in supporting self-regulation and Theory of Mind (ToM), i.e., the development of executive functioning and social cognition (37). Language and EF development thus share protective and risk factors based on the quality of early infant-caregiver interactions (38).

### Socio-Emotional Development

#### Social Synchrony

Social synchrony is considered the core mechanism underlying social skills (39). Synchrony refers to the “dynamic and reciprocal adaptation of the temporal structure of behaviors between interactive partners,”[(40) p3] including the exchange of hormonal, physiological, and behavioral information.

Social synchrony emerges as caregivers begin to synchronize with the infant’s non-verbal communications, augmenting reciprocity, positivity and mutual engagement (41, 42). This continues to evolve throughout childhood and adolescence, “tuning” the social brain for future social interactions (41, 43). Social synchrony is the relational precursor to the development of the neural empathic network, which includes the amygdala, insula, temporal pole and ventromedial prefrontal cortex (43). Callaghan et al. (44) termed this the “neuro-environmental loop” of plasticity, comprising the interaction of early parental care, central nervous system and behavior, in the formation of the amygdala-medial prefrontal cortex network that underlies emotional functioning (44).

Oxytocin has been identified as an important mechanism whereby the attachment experience epigenetically establishes and shapes social development (3, 15). Referred to as the ‘hormone of love or attachment,’ oxytocin promotes physiological and behavioral readiness for parent-infant interactions (45). The caregiver’s oxytocin levels influence his/her behavior, which in turn impacts attachment, as well as the infant’s developing oxytocin systems. Neurobiologically, oxytocin directs the infant to the social micro-behaviors such as eye contact, vocalization, touch and affect, which facilitate social synchrony and reciprocity. This, in turn, increases oxytocin levels in the caregiver and strengthens neural connections in the limbic system responsible for emotion and behavior (45). These core attachment mechanisms underly the development of social and emotional skills central to social communication.

#### Joint Attention

The emergence of the capacity to share or coordinate attention with a social partner, i.e., “joint attention,” is an important milestone in infancy (3, 46). The infant’s initial reciprocal engagement with the caregiver is referred to as “primary intersubjectivity,” i.e., through eye contact, and/or vocalizations in face-to-face interactions, which is considered crucial for social bonding and development of social knowledge (47). Primary intersubjectivity facilitates the development of secondary intersubjectivity, as a continuous process from a dyadic (person-to-person) interaction, to a triadic engagement, which involves joint attention in reference to external objects (48). In this way, shared interactions between the infant and caregiver are precursors of joint attention behaviors (49, 50). This view is consistent with an attachment lens and is rooted in the premise that human nature is innately intersubjective. An alternative view has been espoused by Tomasello and colleagues’ theory of shared intentionality (48, 51, 52). They propose that the capacity for social relatedness is uniquely human and develops as infants engage in behaviors of joint attention, imitative learning, and cooperative action (48, 51–53). Accordingly, the cognitive development of shared intentionality allows the individual intentionality to shift to the shared attention of an external entity (54).

From a neuropsychological perspective, the attuned caregiver-infant interactions ensure that relevant brain systems receive sufficient social input to develop primary intersubjectivity and joint attention skills (55, 56). Joint attention is a key component of the functional development of the neural systems for social cognition and language acquisition, within a dynamic relationship of mutual reinforcement (57). Joint attention is empirically linked to attachment, with insecurely attached children showing less face-to-face attention and less coordinated joint attention to objects, compared to securely attached children (58).

The development of these skills may be negatively impacted by “disorganized” attachment, resulting from environmental care-giving factors such as maltreatment or inconsistent, unresponsive, or fear-evoking caregiving (46). The emergence of joint attention and language are functionally intertwined, rooted in attuned caregiver interactions.

#### Theory of Mind

Theory of Mind (ToM) is fundamental to effective and appropriate social communication (59). In order to communicate socially, it is necessary to have mental representations of the self and others, as well as the language to understand the mental representations and regulate emotions (59, 60). ToM was originally defined by Woodruff and Premack (61) as the ability to understand behaviors using representations of the mental states of oneself and others. ToM is a multi-dimensional construct, which includes cognitive and affective ToM, as well as intrapersonal and interpersonal ToM. Each dimension has differential neuropsychological and neuroanatomical underpinnings and behaviors (60).

These distinctions are important with respect to understanding the relationship between language, ToM and attachment. Most research has focused predominantly on interpersonal cognitive ToM, however, it is important to understand that ToM development starts with intrapersonal ToM, the awareness of one’s own physical and mental world, both at a cognitive and affective level (59). It is the attuned caregiving of the attachment relationship, in the course of primary and secondary intersubjective interactions, that lays the foundations for ToM (59). Through joint attention, infants not only develop shared attention of an object, but also engage in social referencing, i.e., reference other’s perspectives, by interpreting the intents, emotions and perspectives of the caregiver (59). At the same time, ToM facilitates language learning as the caregiver’s words encode the (intrapersonal) infant’s mental states, feelings and direct shared attention, as well as (interpersonal) referential intentions and perspectives of others (60). The capacity of the caregiver to respond to joint attention, is linked with subsequent vocabulary acquisition (62). Thus, language and ToM development have a strong reciprocal connection (63), are integral to social communication (64), and develop in the context of the attachment relationship.

#### Emotion Regulation

Attachment theory is, in a sense, a regulatory theory (65) in that it espouses the development of self-regulation, shifting from an interpersonal (caregiver–child) system of emotion regulation, to an intrapersonal regulation system (66, 67). Emotion regulation, including emotional understanding of self and others, is fundamental for social connections with others (68). The caregiver’s “affect attunement” (39) and “affect synchrony” (69) are based on an “alignment of internal experiences,” and are central to the regulatory processes that promote states of positive arousal, reparative interactions and modulate negative states of arousal (68).

Initial affective communications are non-verbal, i.e., pragmatic and paralinguistic, including tone of voice, gestures, postures, and facial expressions (3) (see Figure 1: SoCom). These paralinguistic social communication behaviors are integral to the development and maintenance of synaptic connections in the right hemisphere, which subserve processing of emotions, stress modulation and self-regulation (17, 65, 67, 70, 71). Additionally, the caregiver’s use of words to describe mental states, verbalize feelings and direct shared attention to the infant’s state of mind, promotes the child’s ability to identify and share emotions (68). This phenomenon of “mentalizing,” helps the infant begin to identify their internal states, fosters a state of resonance, as well as promote language development and its use, to regulate affective states and behavior across the lifetime (65, 72–75). In this sense, the attuned caregiver acts as an external psychobiological regulator (13). Over time, children begin to use language as an executive skill to self-talk in order regulate their emotions and behavior (5, 76–80).

The primary caregiver’s ability to “mentalize” is furthermore predictive of the development of empathy and ToM in the child (81–83). Empathy develops in concert with other cognitive functions, such as emotion regulation and social understanding (84), whereas ToM is fundamental to effective social/pragmatic language skills and is closely intertwined with language development (85). Exposure to rich conversation about emotion and social situations during childhood develops words and meanings about mental states, as well as ToM, which in turn supports pragmatic language development (59). The attuned attachment behaviors thus lay important neurobiological foundations to support self-regulation which is vital for social communication.

### Socio-Linguistic Development

Human infants are born vulnerable and dependent, their survival and ability to thrive depends on successful communication with a caregiver. Language, a fundamental component of social communication, evolves in the context of secure attachment, beginning with non-verbal, paralinguistic communications such as intonations, vocalizations, facial expression, coordinated eye-to-eye messages, and posture. These are processed by the infant’s right hemisphere, interacting with the mother’s right hemisphere, and thereby become imprinted in an IWM. Evidence further suggests an association between language and attachment patterns, as well as between language and caregiver characteristics (86). Infants of caregivers who are responsive to communication signals, such as babbling, attain language milestones earlier than infants of less responsive caregivers (87). The importance of responsive verbal interactions, exposure to a broad range of words and syntactic forms, as well as use of decontextualized language, i.e., talk that extends beyond the here and now, has been empirically documented (88–90). A secure attachment is also associated with better syntax development (87) and overall communication (86, 91). On the contrary, maltreating caregivers interact less, ignore their children more, react infrequently when their children talk, and use less diverse vocabulary and syntactic structures during communication (92–94). These children, in turn, demonstrate delays in syntactic development, reduced vocabulary, poor auditory comprehension skills and pragmatic impairments (95–97). The serve and return exchanges that support infant-caregiver attachment continue beyond infancy, and this prolonged exposure to scaffolding and interactions serves to refine and enrich social communication skills (98).

## Conclusion and Clinical Implications

A developmental neuropsychological perspective offers a valuable framework to understand the interplay of attachment with the development of language, emotion and cognition, which can inform an integrated approach to the assessment of and interventions for social communication. We propose that assessment of individuals with social communication impairments include a review of attachment history, as well as pertinent biopsychosocial and environmental factors. This biopsychosocial profile can inform an individualized intervention plan which supports the child and their relevant social systems, and would be best served by a multidisciplinary team (e.g., speech-language pathology, psychology).

The assessment of social communication impairments should also include screening for possible underlying neuropsychological impairments (e.g., cognitive and linguistic dimensions of ToM, and EFs supporting social skills, such as working memory, inhibition, flexibility). Addressing any neuropsychological impairments concurrently is important to increase the efficacy of social communication intervention. Moreover, in children with communication impairments (e.g., DLD), the language impairment may negatively influence cognitive development, social development and attachment relationships. Assessment and intervention should consider and address both the neurodevelopment of the child as well as their relationships and social systems as this broader lens may help to mitigate adverse social and mental health outcomes.

The neuropsychological implications of early attachment highlight the importance of early prevention which can leverage the neuroplasticity of early years. This early prevention may include support and education to at-risk families, as well as targeted interventions for at-risk infants and children. It is our hope that such an approach will enhance the prevention, assessment and intervention of mental health and social communication disorders.

## Author Contributions

CW-H and LK developed the SoCom model on which the manuscript was based. CW-H scaffolded the manuscript and the early conceptualization. VJ, JK, CW-H, and LK contributed to the literature review, conceptualization and writing of the manuscript, and the editing process. All authors contributed to the article and approved the submitted version.

## Conflict of Interest

The authors declare that the research was conducted in the absence of any commercial or financial relationships that could be construed as a potential conflict of interest.

## Publisher’s Note

All claims expressed in this article are solely those of the authors and do not necessarily represent those of their affiliated organizations, or those of the publisher, the editors and the reviewers. Any product that may be evaluated in this article, or claim that may be made by its manufacturer, is not guaranteed or endorsed by the publisher.
