# Corrigendum: Early attachment and the development of social communication: A neuropsychological approach

**DOI:** 10.3389/fpsyt.2022.944889

**Published:** 2022-07-19

**Authors:** Vibhuti Jethava, Jocelyn Kadish, Lisa Kakonge, Catherine Wiseman-Hakes

**Affiliations:** ^1^York Hills Centre for Children, Youth and Families, Richmond Hill, ON, Canada; ^2^Department of Speech-Language Pathology, University of Toronto, Toronto, ON, Canada; ^3^School of Rehabilitation Science, Speech Language Pathology Program, McMaster University, Hamilton, ON, Canada; ^4^KITE Research Institute, Toronto Rehab-University Health Network, Toronto, ON, Canada; ^5^Holland Bloorview Kids Rehabilitation Hospital, Toronto, ON, Canada

**Keywords:** social communication, social cognition, mental health early attachment and relationships, intervention, assessment neuropsychological approach, infant development

In the original article, the following references were incorrectly inserted and have been removed.

34. Duffy SN, Craddock KJ, Abel T, Nguyen PV. Environmental enrichment modifies the PKA-dependence of hippocampal LTP and improves hippocampus-dependent memory. Learn Mem. (2001) 8:26–34. doi:10.1101/lm.36301

35. Meaney MJ, Aitken DH, Viau V, Sharma S, Sarrieau A. Neonatal handling alters adrenocortical negative feedback sensitivity and hippocampal type II glucocorticoid receptor binding in the rat. Neuroendocrinology. (1989) 50:597–604. doi:10.1159/000125287

38. Tabachnick AR, He Y, Zajac L, Carlson EA, Dozier M. Secure attachment in infancy predicts context-dependent emotion expression in middle childhood. Emotion. (2021) 22:258–69. doi:10.1037/emo0000985

41. Stern JA, Cassidy J. Empathy from infancy to adolescence: an attachment perspective on the development of individual differences. Dev Rev. (2018) 47:1–22. doi:10.1002/imhj.21757

47. Orpinas P. Social Competence. 4th ed. In: Weiner IB, Craighead WE editors. Corsini Encyclopedia of Psychology. (Vol. 4), Hoboken, NJ: John Wiley & Sons, Inc (2010).

In the original article, the following article was not cited

“Hanno E, Surrain S. The direct and indirect relations between self-regulation and language development among monolinguals and dual language learners. *Clin Child Fam Psychol Rev*. (2019) 22:75–79. doi:10.1007/s10567-019-00283-3”

This reference replaces reference number 34 throughout the body of the paper. Corrections have been made to Introduction, “Socio-Cognitive Development,” paragraph two:

“Self-regulation and language serve as important tools in executive control and are interdependent; self-regulation ability has been shown to drive language development, and vice versa (34). Language facilitates self-regulation by serving as a cognitive tool for mental organization, mental representation, and behavioral planning (5, 34)… Further, cognitive flexibility allows for application of variable rules of language, such as multiple meaning words, and pragmatic rules associated with different contexts (34).”

In the original article, the following article was not cited

“Carollo A, Lim M, Aryadoust V, Esposito G. Interpersonal synchrony in the context of caregiver-child interactions: a document co-citation analysis. *Front Psychol*. (2021) 12:701824. doi:10.3389/fpsyg.2021.701824”

This reference replaces reference 39 throughout the body of the paper. Corrections have been made to **Socio-Emotional Development**, “*Social Synchrony*,” paragraph one:

“Social synchrony is considered the core mechanism underlying social skills (39).”

And to **Socio-Emotional Development**, “*Emotion Regulation*,” paragraph one:

“The caregiver's “affect attunement” (39) and “affect synchrony” (72) are based on an alignment of internal experiences, and are central to the regulatory processes that promote states of positive arousal, reparative interactions and modulate negative states of arousal (71).”

In the original article, references 35 and 38 were incorrectly cited in Introduction, “Socio-Cognitive Development,” paragraph two.

Instead of “Further, cognitive flexibility allows for application of variable rules of language, such as multiple meaning words, and pragmatic rules associated with different contexts (35, 38)” it should read “Further, cognitive flexibility allows for application of variable rules of language, such as multiple meaning words, and pragmatic rules associated with different contexts (34).”

In the original article, reference 18 was incorrectly cited. A correction has been made to Introduction, “Socio-Emotional Development,” paragraph two. Instead of “Referred to as the ‘hormone of love or attachment,’ oxytocin promotes physiological and behavioral readiness for parent-infant interactions (18)” it should read “Referred to as the ‘hormone of love or attachment,’ oxytocin promotes physiological and behavioral readiness for parent-infant interactions (45).”

In the original article there was an error. Citations were incorrectly added to Introduction, “Socio-Cognitive,” Paragraph two. Instead of “At the same time, self-regulation enables children to maximize language-learning opportunities (10, 34)” it should read “At the same time, self-regulation enables children to maximize language-learning opportunities.”

Similarly, a citation was incorrectly was added in **Conclusion and Clinical Implications**, Paragraph two. Instead of “Addressing any neuropsychological impairments concurrently is important to increase the efficacy of social communication intervention (3)” it should read “Addressing any neuropsychological impairments concurrently is important to increase the efficacy of social communication intervention.”

The authors apologize for these errors and state that this does not change the scientific conclusions of the article in any way. The original article has been updated.

## Publisher's note

All claims expressed in this article are solely those of the authors and do not necessarily represent those of their affiliated organizations, or those of the publisher, the editors and the reviewers. Any product that may be evaluated in this article, or claim that may be made by its manufacturer, is not guaranteed or endorsed by the publisher.
